# Preventing Atrophic Long-Bone Nonunion: Retrospective Analysis at a Level I Trauma Center

**DOI:** 10.3390/jcm13072071

**Published:** 2024-04-03

**Authors:** Christian Ehrnthaller, Klevin Hoxhaj, Kirsi Manz, Yunjie Zhang, Julian Fürmetz, Wolfgang Böcker, Christoph Linhart

**Affiliations:** 1 Department of Orthopaedics and Trauma Surgery, Musculoskeletal University Center Munich (MUM), University Hospital, LMU Munich, 81377 Munich, Germany; klevin.hoxhaj@campus.lmu.de (K.H.); yunjie.zhang@med.uni-muenchen.de (Y.Z.); julian.fuermetz@med.uni-muenchen.de (J.F.); wolfgang.boecker@med.uni-muenchen.de (W.B.); christoph.linhart@med.uni-muenchen.de (C.L.); 2 Institut für Medizinische Informationsverarbeitung, Biometrie und Epidemiologie (IBE), Medizinische Fakultät, LMU München, Marchioninistr. 15, 81377 München, Germany; manz@ibe.med.uni-muenchen.de; 3 Department of Trauma Surgery, Trauma Center Murnau, Professor-Küntscher-Straße 8, 82418 Murnau am Staffelsee, Germany

**Keywords:** nonunion, long bone, fracture healing, femur, pseudarthrosis, delayed fracture healing, atrophic

## Abstract

**Background**: Among the risk factors for nonunion are unchangeable patient factors such as the type of injury and comorbidities, and factors that can be influenced by the surgeon such as fracture treatment and the postoperative course. While there are numerous studies analyzing unchangeable factors, there is poor evidence for factors that can be affected by the physician. This raises the need to fill the existing knowledge gaps and lay the foundations for future prevention and in-depth treatment strategies. Therefore, the goal of this study was to illuminate knowledge about nonunion in general and uncover the possible reasons for their development; **Methods**: This was a retrospective analysis of 327 patients from 2015 to 2020 from a level I trauma center in Germany. Information about patient characteristics, comorbidities, alcohol and nicotine abuse, fracture classification, type of osteosynthesis, etc., was collected. Matched pair analysis was performed, and statistical testing performed specifically for atrophic long-bone nonunion; **Results**: The type of osteosynthesis significantly affected the development of nonunion, with plate osteosynthesis being a predictor for nonunion. The use of wire cerclage did not affect the development of nonunion, nor did the use of NSAIDs, smoking, alcohol, osteoporosis and BMI; **Conclusion**: Knowledge about predictors for nonunion and strategies to avoid them can benefit the medical care of patients, possibly preventing the development of nonunion.

## 1. Introduction

Bone healing is a long and complex healing process in the body that depends on many different factors [1]. These include individual characteristics, as well as fracture properties. With a good understanding of these factors, fracture healing can be promoted not only qualitatively, but also in terms of time. In rare cases, however, defective or delayed fracture healing occurs. Delayed fracture healing with the subsequent development of nonunion is one of the most difficult conditions to treat and with a great socio-economic impact in trauma surgery patients [2,3].

To date, there is no clear or exact definition of nonunion. There are differences in terms of the period of fracture healing, with the majority of the literature assuming the state of nonunion after 9 months [4]. Ultimately, not only is the exact healing latency important, but also the correlation between the radiological findings and the clinical situation of the patient [5,6,7]. Nonunion is generally divided into three different types:

▪ Hypertrophic nonunion;
▪ Atrophic nonunion;
▪ Infectious nonunion.

Depending on the underlying pathology, the treatment of nonunion must be approached individually and tailored according to each patient and fracture characteristics. The treatment concept consists of the four pillars: radical surgery; soft tissue and bone management; biomechanical stability; and syst./local antibiotic therapy, also known as the “Diamond Concept”, according to Giannoudis et al. [8,9,10]. Since its introduction over a decade ago, this concept has gained wide acceptance in the assessment and planning of nonunion fracture management. This model takes into account the heterogeneity of the underlying physiological and clinical appearance and supports the individual choice of treatment in order to create the best possible biological and mechanical conditions.

Although there are generally accepted principles for fracture treatment which should help minimize the risk for nonunion development [11], the exact cause for the development of nonunion, especially atrophic nonunion, remains unknown.

A high number of studies have attempted to identify the risk factors for the development of nonunion so far [2,7,12,13,14,15]. Most studies have focused on comorbidities and their effect on the development of bone healing delay. Among the generally accepted risk factors are smoking, diabetes, obesity and soft tissue damage, as well as severe fracture classification [13,15]. While the pathophysiological role for these comorbidities and trauma characteristics is relatively clear, these parameters are usually given and not prone to influence by orthopedic surgeons. This study is the first to focus on the traumatological parameters that may contribute to the development of a bone healing delay or nonunion. In daily clinical routine, there is a multitude of theories and opinions regarding which form of treatment is the best and which may contribute to the development of pseudarthrosis. Unfortunately, scientific evidence here is rather limited. Therefore, with this study, we attempted to gain a deeper insight into the true background of the development of nonunion and possibly identify the risk factors which, if considered, could prevent a delay in bone healing.

## 2. Materials and Methods

### 2.1. Study Design

As the study design, a retrospective analysis of patients treated conservatively or surgically for nonunion in the period from 2015 to 2020 at the Department of General, Trauma and Reconstructive Surgery—Campus Großhadern and City Campus was chosen. The patient group was identified using the hospital’s radiological and clinical databases. Firstly, all patients potentially eligible for the study were selected using the corresponding keywords and ICD-10 codes. Then, duplicate entries and all patients with missing records or inadequate imaging were eliminated. In the end, 327 patients in total were included in the study.

### 2.2. Inclusion Criteria

- Atrophic, hypertrophic and infectious nonunion
- Age > 18 years
- Conservative or surgical therapy

### 2.3. Data Collection

After a review of patient records (radiological imaging, surgical protocol, anesthesiologic protocol, patient charts), the following parameters were recorded:

- Age, weight, height, gender;
- Alcohol and nicotine abuse;
- Comorbidities;
- Fracture classification;
- Fracture location;
- Previous surgical/conservative treatments;
- Nonunion type;
- Use of bone supplements, autograft or comminution;
- Postoperative fracture characteristics (anatomic reduction, stability);
- Mechanical complications;
- Wound healing disorders;
- American Society of Anaesthesiologists (ASA) score.

**Figure 1:**
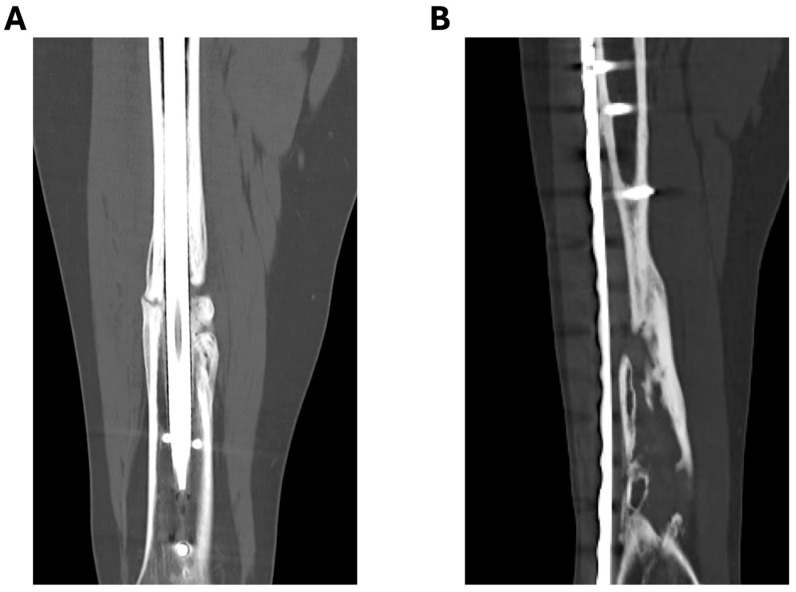
Examples of atrophic femoral nonunions. (**A**) Diaphyseal nonunion after intramedullary nailing; (**B**) diaphyseal nonunion after minimally invasive plate osteosynthesis.

In patients with multiple nonunions, all patient-specific parameters were considered and noted again for each case, even if it was the same patient with another nonunion. Thus, each nonunion including all parameters was evaluated individually, and 167 atrophic (Figure 1), 91 hypertrophic and 69 infectious nonunions were continuously reported in the patient collective.

Postoperative fracture characteristics such as anatomic reduction and the definition of postoperative “stability” were assessed by two independent senior consultants of the trauma department according to postoperative radiographs. “Stability” was defined as the presence of osteosynthesis, which would be stable enough to perform at least partial weight-bearing. This was estimated based on the AO principles (Arbeitsgemeinschaft für Osteosynthesefragen) with respect to reduction, implant size and positioning, number of screws and the overall aspect of the osteosynthesis in regard to the fracture situation.

### 2.4. Statistics

The historical patient data were retrieved from the inpatient database of our hospital (Meona Ltd., Freiburg, Germany) and irreversibly anonymized in a confidential database (Microsoft Excel 2018, Microsoft Corporation, Redmond, WA, USA) before analysis. Our data were processed and analyzed using the statistical program “SPSS” (SPSS Statistics 29, IBM, New York, NY, USA) and “R” (Version 4.2.3, R Project, Vienna, Austria), in compliance with data protection regulations.

We retrospectively recorded the relative frequency of the parameters mentioned for all patients and compared them descriptively to gain a comprehensive insight into the central tendency measures and the dispersion of the variables under consideration. Both the mean and median were calculated for continuous data, and standard deviation was used to quantify the dispersion of the data points around the mean. In addition, we examined the static correlation between different parameters by analyzing linear and logarithmic regressions.

Specifically, logistic regression with a calculation of the odds ratio (OR) with a 95% confidence interval (CI) was used to analyze the incidence of nonunion compared to controls, as well as binary outcome variables. The tests for statistical significance were always performed as “two-tailed”, allowing for a comprehensive assessment of the possible differences or correlations in both directions.

The non-parametric Mann–Whitney U test was used to compare the nonunion group with the control group after testing for normality.

Regarding specific questions, we developed a matched-pair (age and gender) patient collective that served as the control group for nonunion of long tubular bones (humerus, radius, ulna, femur, tibia, fibula). Both pure descriptive analysis and direct statistical comparison were performed. Fisher’s exact test was used to analyze categorical variables between the groups, particularly in the four field tables.

## 3. Results

### 3.1. Descriptive Analysis of the Nonunion Collective

A descriptive analysis of our study collective is shown in Table 1. Three hundred and twenty-seven patients were analyzed, with a wide variety of ages (20–99 years), and predominantly male (60%) patients. Smoking was recorded for almost 30% of the cases, and the most frequent nonunion site was the femur, accounting for 25% of all cases. Atrophic (51%) and 28% hypertrophic nonunions were recorded. Polytrauma and osteoporosis were almost equally represented in 12–13% of the patients. Most often, plate osteosynthesis was performed in 44% followed by intramedullary nailing in 25%. An anatomic reduction was achieved in 61%, and stability was estimated in 75% of cases.

### 3.2. Descriptive Analysis of Atrophic Nonunions

Further, atrophic nonunions of long bones (humerus, radius, ulna, femur, tibia, fibula) were descriptively analyzed (Table 2). No significant differences could be found when compared to the whole nonunion collective described above. In contrast, sex was almost equally distributed. The nonunion site was also more distributed among long bones, with a smaller amount of femur nonunions (34.7%). There were less patients who achieved an anatomic reduction (59%) and stability of the osteosynthesis (70%) postoperatively. Contrary to the complete nonunion collective, we analyzed the use of aspirin (ASS) and non-steroidal anti-inflammatory drugs (NSAIDs). While 9% of the patients were treated with ASS, only 3% used NSAIDs according to medical records.

### 3.3. Atrophic Nonunion vs. Matched-Pair Control Group

To elucidate and highlight the possible pathomechanisms of atrophic long-bone nonunions, a direct statistical comparison of atrophic nonunions and the matched-pair control group was performed (Table 3).

The control group was matched to the nonunion group for age and gender. Like the nonunion group, the control group consisted of 98 patients. The distribution among long bones is shown in Table 3. The femur and tibia were the two most common nonunion sites in the control group, accounting for 34.7% and 23.5%, respectively.

The distributions of the fracture locations are not statistically significantly different between men and women (*p* = 0.72 (Fisher’s exact test)).

### 3.4. Type of Implant

**Figure 2:**
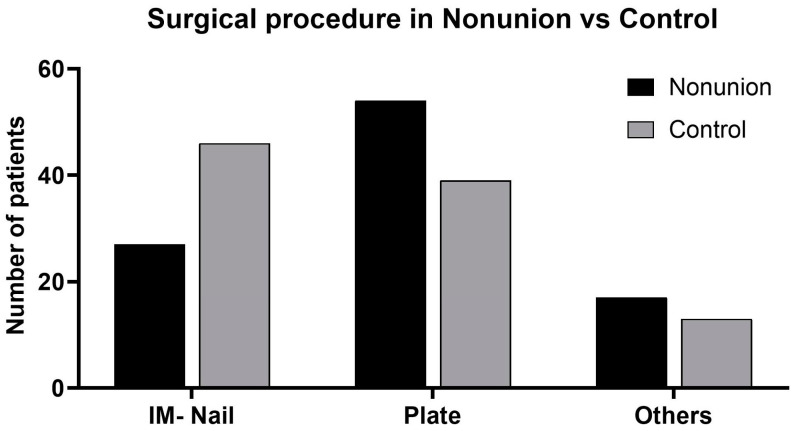
Surgical procedure employed in the nonunion group vs. control group in total numbers.

Intramedullary nailing and plate osteosynthesis were the two most common surgical methods employed (Table 3). If we consider only the two methods, the odds ratio for the development of a nonunion is significantly increased in patients with plate osteosynthesis compared to patients undergoing intramedullary nailing (Figure 2) (OR = 2.36; 95% CI = 1.26, 4.42; *p* = 0.008).

### 3.5. Cerclage Wiring

The use of cerclage wiring was of special interest and was analyzed separately. Hereby, we were able to demonstrate that the use of cerclage wiring corresponds to a lower odds ratio for nonunions, although not significantly (OR = 0.62; 95% CI = 0.32, 1.23; *p* = 0.173).

**Figure 3:**
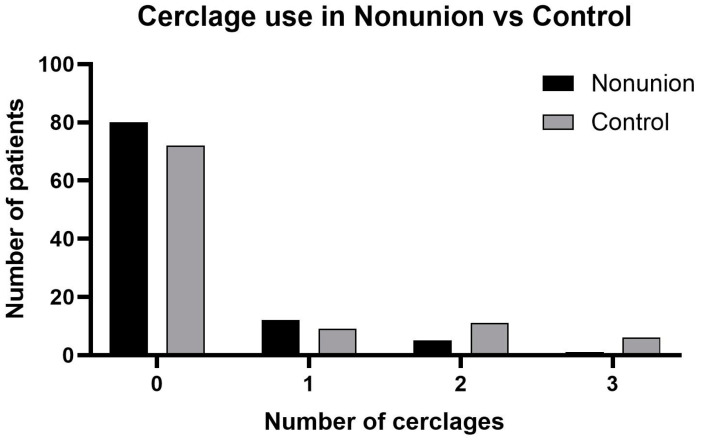
Use of wire cerclages in the nonunion group vs. control group in total numbers.

Further analysis of the number of cerclage wires used showed a different result. Here, the more cerclage wires that were used, the lower the risk for the development of a nonunion was (Figure 3) (OR = 0.66; 95% CI = 0.45, 0.97; *p* = 0.033).

### 3.6. Use of Anti-Inflammatory Drugs

Analysis for the use of acetylsalicylic acid (ASS) (OR = 0.52; 95% CI = 0.22, 1.24; *p* = 0.139) and non-steroidal anti-inflammatory drugs (NSAIDs; ASS excluded) (OR = 0.36; 95% CI = 0.09, 1.38; *p* = 0.14) showed no significant effect on the development of long-bone atrophic nonunion.

### 3.7. Anatomic Reduction/Stability

There is a statistically significantly elevated risk for the development of a nonunion in patients where an anatomic reduction (*p* < 0.0001) or stability after osteosynthesis could not be obtained (*p* < 0.0001).

### 3.8. Polytrauma/Osteoporosis/Vitamin D/Smoking/Alcohol Abuse

**Figure 4:**
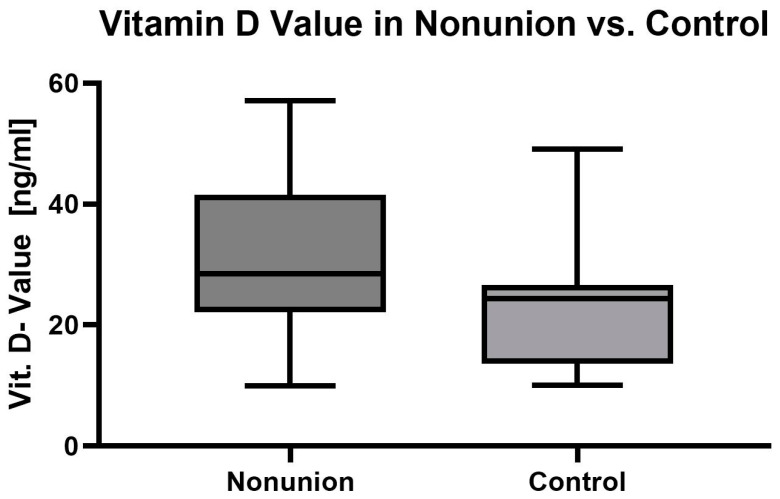
Vitamin D value in ng/mL in the nonunion vs. control group.

When comparing atrophic long-bone nonunion with the control cases, one could not find any significant changes when looking at factors such as polytrauma, osteoporosis, vitamin D value (Figure 4), smoking and alcohol abuse (for details, see Table 3).

## 4. Discussion

This study was the first to show the factors which could have an influence on the development of delayed fracture healing or nonunion during surgical fracture osteosynthesis. Unfortunately, previous studies have often been limited to comorbidities and patient factors that are not within the surgeon’s sphere of influence [2,6,7,13,14,15]. Due to the lack of evidence in this area, a variety of opinions and theories on the development of nonunions have developed at clinics and institutions, which, on closer examination, do not stand up to evidence-based scrutiny.

Besides obvious reasons such as bad primary surgical treatment with large gaps or failure to achieve sufficient stability, patients even developed nonunion one would have never thought possible at times.

In line with the available literature, this study confirmed that factors such as anatomical reduction and stability have a significant influence on the development of nonunion [13]. Other factors, which are generally assumed to be risk factors, could not be confirmed in this study. For example, there was no significant increase in the risk for smoking, polytrauma, osteoporosis and BMI. This is quite surprising given that, as already mentioned, many studies describe these factors as risk factors for the development of nonunion [2,3,7,12,13,14,15].

Smoking is cited as a risk factor in almost all studies; however, there are also several studies that have seen no correlation, as in the present study [16]. Another controversial example is BMI and age. The data situation here is very heterogeneous. In some cases, both advanced age [17] and BMI [13] are seen as risk factors, but in others, there is a clear trend to the contrary, with the frequency of nonunion even decreasing in older age [14].

One reason for the strong fluctuations in the literature could be the different study designs. Almost all available studies do not differentiate between the pathogenesis of nonunion, meaning that the study collectives contain a mixture of infectious pseudarthroses with atrophic and hypertrophic pseudarthroses. Since the underlying problem is completely different depending on the type of nonunion, it is obvious that the risk factor predictors also differ, and it is difficult to analyze them. This is the reason why this study mainly deals with atrophic nonunions of long tubular bones. In our view, the bias of an inhomogeneous study collective can be reduced as much as possible. While we also looked at population-based predictors in this study, the focus was set on factors that can be influenced by the treating physician and that could have an impact on the development of pseudarthrosis as part of its clinical treatment.

The most urgent question in the case of acute shaft fractures of long tubular bones is in regard to the method of fixation to be selected.

While some fractures clearly indicate the use of either an intramedullary (mainly diaphyseal fractures) or an extramedullary (mainly metaphyseal proximal or thistle) implant, the data situation for proximal or distal shaft fractures at the meta-diaphyseal junction is less clear [18,19]. In this case, the choice of implant is primarily dependent on the personal preferences of the surgeon or on the usability and availability of implant types. The present study showed that the use of plate osteosynthesis is a risk factor for the development of a nonunion across all atrophic nonunions of long tubular bones. Some studies also showed evidence for a shorter bone union time [19], whereas others were not able to detect any significant differences [18]. The reason for a possible higher nonunion time after plate osteosynthesis could lie in the implantation technique. Although often implanted, MIPO (minimally invasive plate osteosynthesis) results in a bigger anatomical reduction compared to intramedullary implants usually achieved through direct open visualization. This leads to increased soft tissue trauma and disruption of the environment around the fracture. In line with AO guidelines and biological osteosynthesis principles [20], the fracture area is left untouched with intramedullary implants, and although an anatomical reduction is often not achievable, this appears to be a more advantageous approach in terms of delaying healing. In order to objectify the impact of surgical trauma in patients, a paper dealing with sterile inflammation after surgical therapy was published only recently. Here, the elevated inflammatory impact of additional surgical trauma from open reduction was highly significant [21]. Besides short-term side effects such as prolonged hospital stay and blood loss, it is tempting to speculate that this additional sterile inflammation might also play a role in nonunion development.

An important and very heterogeneously discussed point in everyday clinical practice is the use of cerclage and its role or disadvantage in bone healing. Many surgeons are of the opinion that by tightening and compressing the periosteum, cerclage wires could lead to reduced blood flow, thus preventing fracture healing. The data on this are very controversial. While some studies assume a delay in healing [22,23], others have even found an acceleration in healing times [24] or no significant difference [25]. A major advantage of using cerclage is improvement in the anatomical position of the fracture fragments in relation to each other and the resulting greater stability [23,25], which can have an effect not only on blood flow but also on the success of fracture healing. However, no predictors for the development of nonunion using cerclage wires were found in this study. On the contrary, the risk actually decreased significantly the more cerclage was used. As already mentioned, one possible reason for this could be the more precise reduction and thus the more favorable biomechanical conditions achieved.

Another point that is treated inconsistently in clinical routine is the use of NSAIDs during the postoperative phase. Based on animal studies showing an important role for COX-2 in fracture healing [26], the postoperative pain management protocol has been changed in many hospitals, and NSAIDs have been avoided whenever possible [27]. Meta-analyses and epidemiologic studies show a heterogeneous picture. While some meta-analyses have shown no negative effect on fracture healing [28,29], more recent observational studies have shown that selective COX-2 inhibitors were associated with a delay in fracture healing [30], whereas nonselective COX-2 inhibitors showed no negative effect. In our study, no increase in the nonunion rate was found with the use of NSAIDs; however, we did not analyze selective COX-2 and non-selective COX-2 inhibitors separately because selective COX-2 inhibitors were generally not used at our hospital.

### Limitations

Although the investigated collective represents a realistic study group of patients suffering nonunion, some limitations have to be taken into account. Even though a large collective was analyzed, and significant differences were observed between the groups, the number of patients in the subgroups is limited. Matched-pair analysis was carried out with one patient each, and a higher number could have ruled out some selection bias. Due to the retrospective study design, there was no evaluation of the definitive outcome of the chosen treatment regime and no evaluation of PROMs (patient-reported outcome measures). Some parameters, such as “stability” or “anatomic reduction”, are dependent on the examiner and therefore prone to bias. To minimize this risk, these subjective factors were evaluated independently by two senior orthopedic surgeons. Lastly, missing information about the patients in the clinical chart might have altered the results.

## 5. Conclusions

Nonunion, especially atrophic nonunion, is one of the greatest challenges in orthopedic surgery. For the patient, the development of nonunion represents a major impairment and leads to prolonged medical treatment with consequent physical disability and high socioeconomic burden due to medical leave and treatment costs. In addition to the given factors such as type of injury, comorbidities, soft tissue damage and concomitant injuries, the factors prone to influence especially give surgeons the opportunity to avert complications. This study therefore sought to evaluate such factors to possibly minimize the risk for nonunion development.

Besides obvious surgical goals such as anatomic reduction and stable fixation, it is of note that plate osteosynthesis increases the risk for nonunion development, whereas the use of cerclage wires does not seem to affect the development of nonunion in a negative way. Besides other important factors, the often-highlighted negative role of NSAIDs in nonunion development could also not be confirmed. This study is a very good example that in everyday clinical practice, there are many opinions and hospital treatment standards that are intended to help prevent complications such as nonunion, but their scientific background is often not given, or the evidence is scarce. In this regard, consideration of the results presented here could help prevent the development of atrophic nonunion in some patients.

## Figures and Tables

**Table 1:** Descriptive analysis of the whole nonunion collective.

Factor	Total Numbers	Percentage
**Age (years)**	20–99 (Mean 57.8)	
**Sex (male)**	195	59.6
**BMI**	17–66 (Mean 26.5)	
**Alcohol abuse**	29	8.9
**Smoking**	93	28.4
**Polytrauma**	39	11.9
**ASA score**		
1	50	15
2	144	44
3	112	34
4	4	1
**Osteoporosis**	43	13.1
**Anti-osteoporotic treatment**	34	10.4
**Nonunion site**		
Femur	83	25
Tibia	71	22
Humerus	33	10
Clavicula	27	8
Foot	21	6
Hand	20	6
Radius	17	5
Ankle	14	4
Fibula	12	4
Ulna	10	3
Dens axis	8	2
Os pubis	5	2
Patella	4	1
Scapula	2	1
**Type of nonunion**		
Atrophy	167	51.1
Hypertrophy	91	27.8
infectious	69	21.1
**Bone substitute/autograft**		
At first surgery	12	3.7
At nonunion surgery	146	44.6
**Cerclage wiring (total number)**		
1	31	9.5
2	11	3.4
3	1	0.3
4	1	0.3
5	0	0
6	1	0.3
7	1	0.3
**Anatomic Reduction**	199	60.9
**Stability**	245	74.9
**Implant removal**	47	14.3
**Soft tissue damage**		
Closed	214	65.4
Grade I closed	30	9.2
Grade II closed	21	6.4
Grade III closed	1	0.3
Not specified	162	49.5
Open	36	11
Grade I open	4	1.2
Grade II open	15	4.5
Grade III open	17	5.1
No data	77	23.5
**Type of osteosynthesis**		
K-wires	5	1.5
External fixator	10	3.1
No data	14	4.3
Conservative	27	8.3
Intramedullary nail	90	27.5
Plate	146	44.6
Cerclage wiring	4	1.2
Screw	24	7.3
Joint replacement	7	2.1

**Table 2:** Descriptive analysis of the atrophic nonunion collective.

Factor	Total Numbers	Percentage
**Age**	22–100 (Mean 63)	
**Sex (male)**	47	48
**BMI**	17.3–66.1 (Mean 24.5)	
**Alcohol abuse**	7	7.1
**Smoking**	18	18.4
**Polytrauma**	14	14.3
**ASA score**	1–4 (Mean 2)	
**Osteoporosis**	15	15.1
**Anti-osteoporotic treatment**	11	11.1
**ASS**	9	9.1
**NSAIDs**	3	3.1
**Vitamin D (ng/mL)**	9.9–57.1 (Mean 24.45)	
**Nonunion site**		
Femur	34	34.7
Tibia	22	22.4
Humerus	17	17.3
Radius	12	12.2
Ulna	6	6.1
Fibula	3	3.1
Tibia, fibula	2	2.1
Radius, ulna	2	2.1
**Bone substitute/autograft**		
At primary surgery	4	4.1
At nonunion surgery	47	47.9
**Cerclage wiring (total number)**		
1	12	12.2
2	5	5.1
3	1	1.1
Anatomic reduction	58	59.1
Stability	69	70
Implant removal	12	12.1
**Soft tissue damage**		
Closed	68	69.4
Grade I closed	11	11.2
Grade II closed	8	8.1
Not specified	49	50
Open	16	16.3
Grade I open	2	2
Grade II open	3	3.1
Grade III open	4	4
No data	21	21.4
**Type of osteosynthesis**		
K-wires	2	2.1
External fixator	1	1.1
No data	1	1.1
Conservative	3	3.1
Intramedullary nail	27	27.5
Plate	54	55.1
Plate + Intramedullary nail	1	1.1
Screw	6	6.1
Joint replacement	3	3.1

**Table 3:** Results from the analysis of atrophic nonunion vs. matched-pair control group.

Parameter	Nonunion No. (%)	Control No. (%)	*p*-Value
Sex (male)	47 (48.0%)	46 (46.9%)	0.99
Age (years)	64 (46–79)	64 (28–78)	0.88
BMI (kg/m^2^)	24.5 (22.5–26.9)	25.7 (22.4–28.6)	0.16
Alcohol abuse	7 (7.1%)	12 (12.2%)	0.33
Smoking	18 (18.4%)	29 (29.6%)	0.09
Polytrauma	14 (14.3%)	18 (18.4%)	0.56
Used implant			
Intramedullary nail	27 (27.6%)	46 (46.9%)	0.021
Plate	54 (55.1%)	39 (39.8%)	
Other	17 (17.3%)	13 (13.3%)	
Use of cerclage wires	18 (18.4%)	26 (26.5%)	0.23
Total number of cerclage wires			
0	80 (81.6%)	72 (73.5%)	0.08
1	12 (12.2%)	9 (9.2%)	
2	5 (5.1%)	11 (11.2%)	
3	1 (1.0%)	6 (6.1%)	
Anatomic reduction			
Yes	58 (59.2%)	88 (89.8%)	<0.0001
No data	7 (7.1%)	0 (0.0%)	
Conservative treatment	3 (3.1%)	0 (0.0%)	
No	30 (30.6%)	10 (10.2%)	
Stability			
Yes	69 (70.4%)	97 (99.0%)	<0.0001
No data	7 (7.1%)	0 (0.0%)	
Conservative treatment	3 (3.1%)	0 (0.0%)	
No	18 (18.4%)	1 (1.0%)	
Vitamin D (ng/mL)	26.8 (15.6–36.7)	18.5 (11.2–26.4)	0.09
Osteoporosis	15 (15.3%)	17 (17.3%)	0.85
Anti-osteoporotic treatment	11 (11.2%)	14 (14.3%)	0.67
Implant removal	12 (12.2%)	27 (27.6%)	0.007
ASA score (mean)	2 (2–3)	2 (2–3)	0.82
NSAIDs	3 (3.1%)	8 (8.2%)	0.21
ASS	9 (9.2%)	16 (16.3%)	0.19

## Data Availability

The data presented in this study are available on request from the corresponding author. The data are not publicly available due to privacy reasons.
